# A method for modelling GP practice level deprivation scores using GIS

**DOI:** 10.1186/1476-072X-6-38

**Published:** 2007-09-06

**Authors:** Mark Strong, Ravi Maheswaran, Tim Pearson, Paul Fryers

**Affiliations:** 1 Rotherham Primary Care Trust, Oak House, Moorhead Way, Bramley, Rotherham, S66 1YY, UK; 2 Public Health GIS Unit, School of Health and Related Research, The University of Sheffield, Regent Court, 30 Regent Street, Sheffield S1 4DA, UK; 3 Public Health Intelligence Unit, Doncaster Primary Care Trust, White Rose House, Ten Pound Walk, Doncaster, DN4 5DJ, UK

## Abstract

**Background:**

A measure of general practice level socioeconomic deprivation can be used to explore the association between deprivation and other practice characteristics. An area-based categorisation is commonly chosen as the basis for such a deprivation measure. Ideally a practice population-weighted area-based deprivation score would be calculated using individual level spatially referenced data. However, these data are often unavailable. One approach is to link the practice postcode to an area-based deprivation score, but this method has limitations. This study aimed to develop a Geographical Information Systems (GIS) based model that could better predict a practice population-weighted deprivation score in the absence of patient level data than simple practice postcode linkage.

**Results:**

We calculated predicted practice level Index of Multiple Deprivation (IMD) 2004 deprivation scores using two methods that did not require patient level data. Firstly we linked the practice postcode to an IMD 2004 score, and secondly we used a GIS model derived using data from Rotherham, UK. We compared our two sets of predicted scores to "gold standard" practice population-weighted scores for practices in Doncaster, Havering and Warrington. Overall, the practice postcode linkage method overestimated "gold standard" IMD scores by 2.54 points (95% CI 0.94, 4.14), whereas our modelling method showed no such bias (mean difference 0.36, 95% CI -0.30, 1.02). The postcode-linked method systematically underestimated the gold standard score in less deprived areas, and overestimated it in more deprived areas. Our modelling method showed a small underestimation in scores at higher levels of deprivation in Havering, but showed no bias in Doncaster or Warrington. The postcode-linked method showed more variability when predicting scores than did the GIS modelling method.

**Conclusion:**

A GIS based model can be used to predict a practice population-weighted area-based deprivation measure in the absence of patient level data. Our modelled measure generally had better agreement with the population-weighted measure than did a postcode-linked measure. Our model may also avoid an underestimation of IMD scores in less deprived areas, and overestimation of scores in more deprived areas, seen when using postcode linked scores. The proposed method may be of use to researchers who do not have access to patient level spatially referenced data.

## Background

A measure of socioeconomic deprivation assigned to the patient population registered with a general practice can be used to explore the association between deprivation and other practice characteristics, such as disease prevalence or quality of care. This then allows questions of equity to be addressed, for example, how are resources distributed in relation to the need for them? There are two commonly used methods for calculating practice level deprivation found in the literature, both of which are based on small-area deprivation measures such as the Townsend index [1] or the Index of Multiple Deprivation [2]. The first method for calculating practice level deprivation takes spatially referenced patient level data and calculates a mean score, weighted for the proportion of the practice population living within each small area (see for example [3-5]). For the purposes of this paper we shall refer to this method as the "gold standard", although we recognise that it has limitations. The second method uses only the location of the practice building itself and links the practice to the score assigned to the small area in which it resides (see for example [6-8]).

The difficulty with the gold standard practice population-weighted method is the need for patient level geographical data. Although these data are used routinely within NHS Primary Care Organisations, they are not easily accessible to researchers working in other parts of the NHS, or in the academic sector [9]. The postcode-linked method is more straightforward, in that it does not require such data, but makes the assumption that the deprivation score associated with the small area in which the practice resides provides a valid proxy for the socioeconomic deprivation experienced by the practice population as a whole. Given that the majority of a practice's registered patient population live in areas surrounding the practice that are likely to have deprivation scores different to that of the area in which the practice is located, this assumption is questionable. We have previously shown than the postcode-linked method will tend to underestimate an association between deprivation and another practice level variable such as mortality [10].

An alternative approach, which we propose, is to model the population-weighted deprivation measure based on an assumed spatial distribution of patients around a "typical" practice, rather than the true spatial distribution for which the data are unavailable. This study aimed to develop such a model using spatially referenced patient level data from Rotherham, UK, and then to test the model's ability to predict practice level population-weighted IMD 2004 scores in three other UK districts: Doncaster, Warrington and the London borough of Havering.

## Methods

### Model construction using Rotherham Primary Care Trust data

In January 2006 there were 39 general practices that contracted with Rotherham Primary Care Trust, with a total registered population of 253,417. Of these registered patients, 246,574 lived within the broadly coterminous Rotherham Local Authority area. One of the 39 Rotherham practices was a small specialist practice providing care for asylum seekers and homeless people and was removed from this analysis. Twenty-five of the remaining 38 practices had a single main surgery building, nine had one branch surgery and four had two branch surgeries. The total number of practice sites was therefore 55 and the total number of Rotherham-resident patients registered with these practices was 245,107.

We constructed 0.1 mile (0.16 km) width concentric ring buffer zones around the practice site postcode centroid for each of the 55 practice sites using geographical information systems (GIS) methods [11]. For every practice we then calculated the proportion of their registered patients whose postcode centroid lay within each of the 0.1 mile concentric ring buffers. Patients are registered at a practice rather than at an individual surgery site, so we made the assumption that patients would attend the nearest site if a practice had one or more branch surgeries. We then calculated the mean of the practice population proportions living within each concentric ring buffer for the 55 practice sites.

Initial examination of the relationship between the proportion of the registered practice population registered per unit area and distance from the practice suggested that an exponential decay function would fit the data. This is in line with previous published literature (e.g. see [12] or [13]), and has a firm theoretical underpinning [14]. We therefore fitted a curve of the general form:

*p*/*A *α exp (-*d*)

i.e. the proportion of the registered population (*p*) per unit area (*A*) is proportional to the exponential decay of distance from the practice (e^-*d*^). Since the areas of concentric ring buffers of equal width are proportional to their distance from the central point, (i.e. *A *α *d*) [14], equation 1 can be expressed as follows:

*p *= **s**_**1 **_*d*. exp (- **s**_**2 **_*d*)

Where **s**_**1 **_and **s**_**2 **_are scaling constants.

We used SigmaPlot 9.0 to fit a general function of this form to the Rotherham practice population proportion versus distance data, and so determine the scaling constants [15]. The resulting function was then taken to describe the proportion of the total registered practice population (*p*) at distance (*d*) from the "average" Rotherham practice site.

### Using the model to predict deprivation scores in the absence of patient level data

We used the Rotherham derived practice population distance function to predict practice level Index of Multiple Deprivation (IMD) 2004 scores for practices for which we had no patient level data. We chose for our study the practices within the following three districts: Doncaster, Warrington and the London borough of Havering. These districts were selected because we had access to published or unpublished "gold standard" practice population-weighted IMD 2004 scores (i.e. scores calculated using patient level spatially referenced data) necessary to test our "modelled" scores. We considered the three districts separately in order to determine the performance of the model in areas with different social and spatial characteristics. Researchers may wish to calculate a deprivation measure for practices within a single district, and we wished to know whether there was likely to be a significant "district" effect.

IMD 2004 is a composite deprivation index containing seven domains: income, employment, health and disability, education, housing, environment and crime. The IMD 2004 is published at Lower Layer Super Output Area (LSOA) level [16] for the whole of England in a downloadable table [17].

We modelled the practice population-weighted IMD 2004 deprivation score as follows. We first calculated the Euclidean distances between the practice building postcode centroid and the centroids of all the LSOAs within the PCT using the Spider Graph function in MapInfo 8.0 [11]. We then estimated the proportion of the practice population living in each LSOA by applying the Rotherham derived distance function. Finally, we calculated the mean of the LSOA IMD 2004 scores, weighted by the modelled practice population proportion multiplied by the LSOA population. LSOA populations were obtained from the 2001 Census via Casweb [18].

Where a practice had a branch surgery in addition to the main surgery we calculated an IMD 2004 score for each practice site using the same method above. Since patients in the UK are registered with a practice, rather than a surgery site there is no way of obtaining data for the distribution of patients between surgery sites. Similar studies to ours that have examined health care accessibility have assigned equal waiting to main and branch surgeries [19,20]. We therefore made the assumption that equal numbers of patients would attend each site, and the overall practice deprivation score was taken as the simple average of the scores for the practice sites. We explored the performance of the model under a range of different main-branch surgery population weightings in a simple sensitivity analysis.

### Calculating simple practice postcode-linked scores

Our model will only be useful if it provides significant benefit over simply linking the practice postcode to an IMD2004 score via the LSOA in which the practice resides. We calculated these scores as follows. For each of the practices in Doncaster, Havering and Warrington we used the All Fields Postcode Directory (now known as the National Statistics Postcode Directory) [21], to link the postcodes of the main surgery building and any branch surgeries to the LSOAs in which the postcode centroids were located, and hence to IMD 2004 scores. For practices with one or more branch surgeries we calculated the average of the IMD 2004 scores linked to the main and branch surgeries.

### Measuring agreement between deprivation scores

We obtained practice population-weighted IMD 2004 score data for the three districts from published or unpublished sources. The data set for Doncaster was calculated as part of routine PCT work but is as yet unpublished; data for Warrington and Havering were obtained from electronically published sources [22,23]. Each data set consisted of the mean IMD 2004 score, weighted for the proportion of the registered practice population living in each LSOA. We refer to these data as the "gold standard" practice level deprivation scores.

We examined the agreement between the gold standard scores and the simple postcode-linked scores, and between the gold standard scores and our modelled population-weighted scores using Bland and Altman's method for measuring agreement [24]. In this method the differences in pairs of scores (i.e. in our case, predicted score - gold standard score) are plotted against the means of the pairs of scores (i.e. (predicted score + gold standard score)/2). We calculated and plotted the mean of the differences in scores for each dataset and the 95% "limits of agreement" (i.e. mean of the differences +/- 1.96 × standard deviation (SD) of the differences). We also calculated 95% confidence intervals for each of the above measures. The resulting chart illustrates the overall agreement between the two measures as well as highlighting any systematic bias in the predicted values.

If predicted scores are in perfect agreement with the gold standard scores the differences between the pairs will all be zero, and the plot will show a series of points along a horizontal straight line. The degree to which the differences deviate from zero gives an indication of the level of disagreement between the predicted and the gold standard scores. The mean of the differences between predicted and gold standard scores tells us whether, on average, the method for predicting scores over- or under-estimates the gold standard population weighted score. The approximate normality of the data allows us to test the hypothesis of no bias using the relatively robust paired t-test [25].

Examining the relationship between the individual differences in pairs of scores and their means is also informative. If a positive relationship exists between the differences in scores and their means, this suggests that deprivation is being overestimated in more deprived areas relative to less deprived areas (likewise the opposite holds if a negative relationship exists). The presence or absence of a linear relationship can be examined by calculating the correlation coefficient [25].

If two different methods for predicting scores are both unbiased then the method that leads to the lesser variability will be the more useful. We tested the hypothesis that the two methods (postcode linking and GIS modelling) had the same variability in predicting the "gold standard" scores using the Fligner-Killeen test for homogeneity of variances within R 2.5.0 [26]. This non-parametric method was chosen since the parametric F-test for homogeneity of variance is too sensitive to the deviations from normality seen in our data.

We compared the two different methods in an overall analysis combining practices from the three districts, and for the three districts separately. The district level analyses allowed us to determine the performance of the model for a relatively small number of practices within a defined area. We did this because researchers may wish to calculate a deprivation measure for practices within a single district, and we wished to know whether there was likely to be a significant "district" effect.

## Results

### Model construction

**Figure 1:**
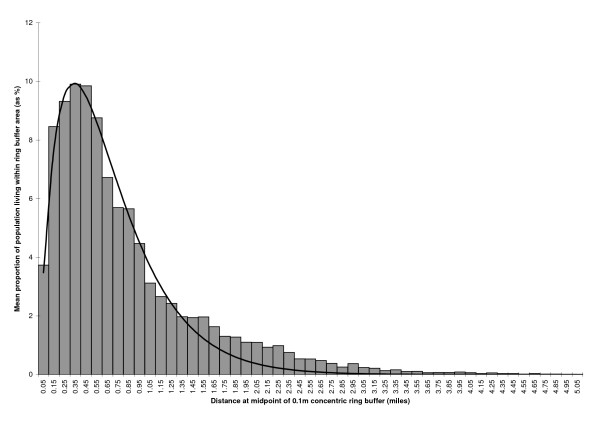
The mean proportion of patients living within 0.1 mile concentric ring buffers by distance from practice (data from 55 Rotherham practice sites). Fitted curve represents equation 2 in text (adj R-square = 0.98).

The 90% effective catchment area (the area that encompasses 90% of the practice population) for the 55 practice sites ranged from 0.8 miles (1.28 km) to 3.3 miles (5.28 km) with a mean of 1.7 miles (2.72 km). (Figure 1) shows the distribution of the mean population proportions by distance from all 55 Rotherham practice sites used to construct the model, along with the fitted exponential decay function curve.

The scaling parameters that allowed the best fit of the exponential decay model to the Rotherham data were **s**_1 _= 80.6822 and **s**_2 _= 2.9844. The adjusted R squared for goodness of fit was 0.98.

### Measurement of agreement between deprivation scores

Population-weighted deprivation scores derived from patient level spatially referenced data were available for 46 practices in Doncaster [unpublished source], 53 practices in Havering [22] and 26 practices in Warrington [23]. We refer to these data as the "gold standard" population-weighted deprivation scores.

**Figure 2:**
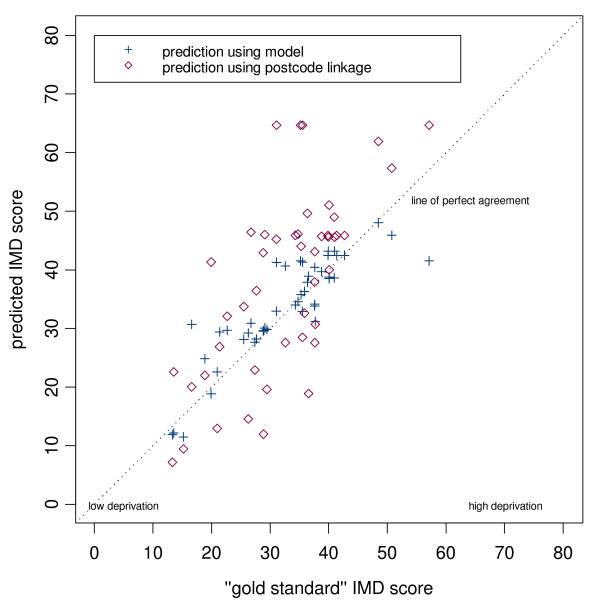
Scatter plot of predicted IMD score (modelled and postcode linked) versus gold standard score for Doncaster practices.

**Figure 3:**
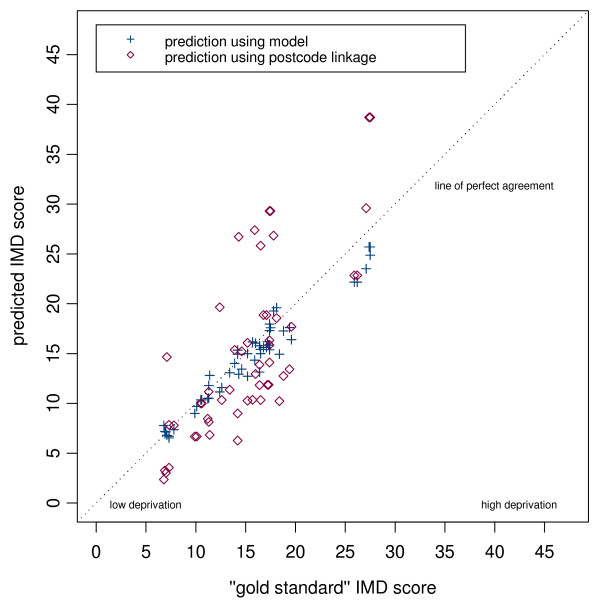
Scatter plot of predicted IMD score (modelled and postcode linked) versus gold standard score for Havering practices.

**Figure 4:**
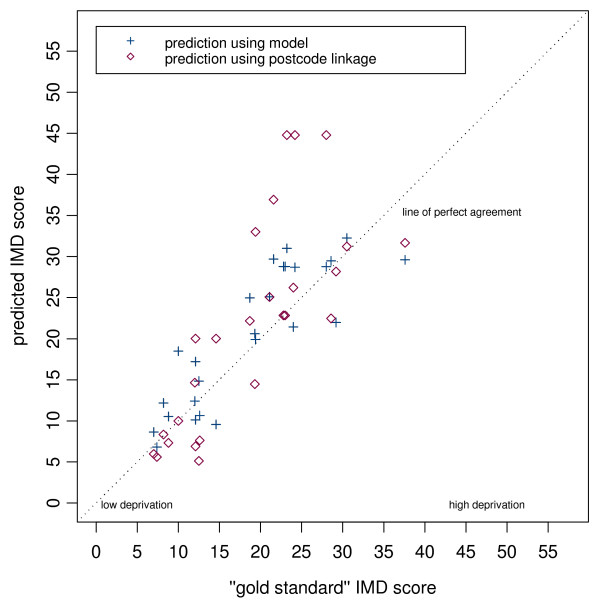
Scatter plot of predicted IMD score (modelled and postcode linked) versus gold standard score for Warrington practices.

Scatter plots of the gold standard population-weighted scores against our simple practice postcode-linked measure, and of the gold standard scores against our modelled population-weighted scores are shown for each district in (Figures 2, 3 and 4). On each plot the dashed diagonal line represents the "line of perfect agreement", along which points would lie if the methods produced identical results.

**Figure 5:**
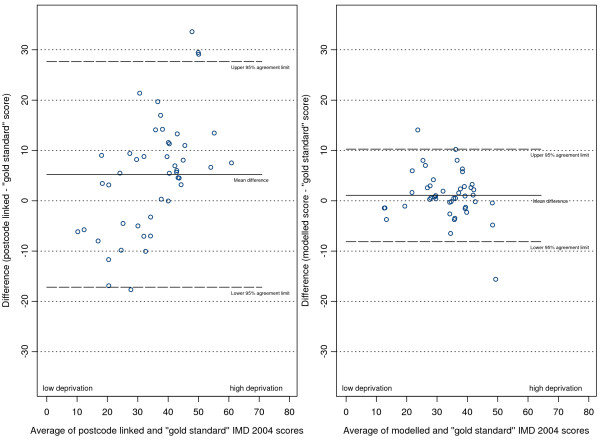
Differences in IMD 2004 scores (predicted score - gold standard score) against their mean for Doncaster practices.

**Figure 6:**
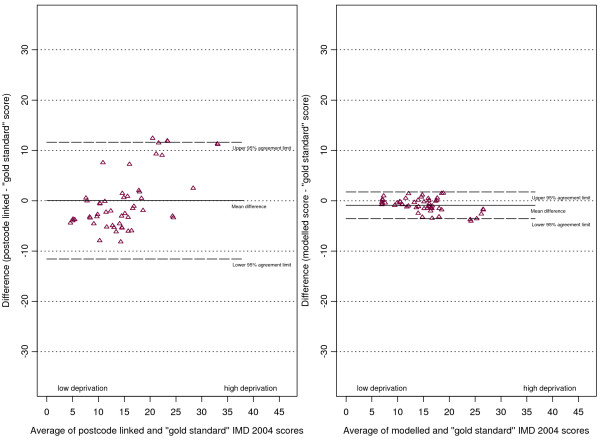
Differences in IMD 2004 scores (predicted score - gold standard score) against their mean for Havering practices.

**Figure 7:**
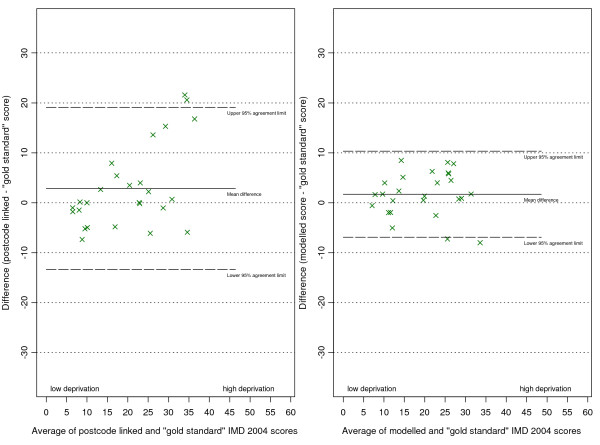
Differences in IMD 2004 scores (predicted score - gold standard score) against their mean for Warrington practices.

**Figure 8:**
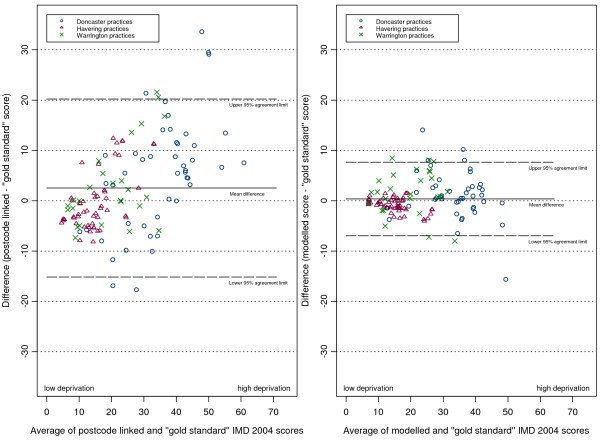
Differences in IMD 2004 scores (predicted score - gold standard score) against their mean for all practices.

The plots of differences in scores against the score means (as per the Bland and Altman method [24]) are shown in (figures 5, 6, 7 and 8). The mean of the differences is shown, along with the 95% "limits of agreement" (see also Table 1). These limits are set at 1.96 standard deviations either side of the mean and give an indication of the dispersion of the data. For all point estimates we calculated 95% confidence intervals and we tested the hypothesis that the mean of the differences was zero (i.e. that the method was unbiased) using a paired t test (significant results are labelled in the table with an asterisk).

**Table 1:** Mean difference and 95% limits of agreement between postcode-linked method and gold standard population weighted method, and between modelling method and gold standard population weighted method.

**District**	**Number of practices**		**Postcode-linked score – gold standard score comparison**	**Modelled score – gold standard score comparison**
Doncaster	46	Mean difference (95% CI)	5.24 * (1.84,8.63)	1.07 ^ns ^(-0.33,2.46)
		Upper limit of agreement (95% CI)	27.65 (21.85,33.45)	10.26 (7.88,12.64)
		Lower limit of agreement (95% CI)	-17.17 (-22.97,-11.37)	-8.13 (-10.51,-5.75)
Havering	53	Mean difference (95% CI)	0.03 ^ns ^(-1.60,1.66)	-0.90 * (-1.28,-0.53)
		Upper limit of agreement (95% CI)	11.65 (8.86,14.44)	1.76 (1.12,2.40)
		Lower limit of agreement (95% CI)	-11.59 (-14.38,-8.80)	-3.57 (-4.21,-2.93)
Warrington	26	Mean difference (95% CI)	2.87 ^ns ^(-0.48,6.22)	1.69 ^ns ^(-0.09,3.47)
		Upper limit of agreement (95% CI)	19.12 (13.40,24.85)	10.32 (7.28,13.35)
		Lower limit of agreement (95% CI)	-13.39 (-19.11,-7.66)	-6.93 (-9.97,-3.90)
All three districts combined	125	Mean difference (95% CI)	2.54 * (0.94,4.14)	0.36 ^ns ^(-0.30,1.02)
		Upper limit of agreement (95% CI)	20.23 (17.57,22.83)	7.67 (6.57,8.77)
		Lower limit of agreement (95% CI)	-15.15 (-17.50,-12.50)	-6.95 (-8.04,-5.85)

* – mean difference significantly different from zero (paired t test p < 0.01)

ns – mean difference not significantly different to zero (paired t test p >= 0.05)

In the combined three-district analysis the postcode linkage method, on average, overestimated the gold standard IMD scores by 2.54 points (95% CI 0.94 to 4.14), whereas our modelling method showed no such bias (mean difference 0.36, 95% CI -0.30 to 1.02). For the subgroup of Doncaster practices the postcode linkage method overestimated the gold standard IMD scores (mean difference 5.24, 95% CI 1.84 to 8.63), while the modelling method showed no bias (mean difference 1.07, 95% CI -0.33 to 2.46). In Havering our modelling method underestimated the gold standard scores by a small but statistically significant amount (mean difference -0.90, 95% CI -1.28 to -0.53), whereas the postcode linkage method showed no bias (mean difference 0.03, 95% CI -1.60 to 1.66). When predicting scores for Warrington neither method showed bias.

There was a systematic tendency for the postcode-linked method to underestimate the gold standard score in less deprived areas, and overestimate it in more deprived areas. This is apparent in the positive linear relationships shown in (figures 5, 6, 7 and 8) (see table 2 for correlation coefficients). Our modelling method showed no such linear relationship when predicting scores in Doncaster and Warrington (figures 5 and 7), or in the combined analysis (figure 8). In Havering there was a moderate negative correlation between the differences in scores and their mean, suggesting that our method underestimated scores at higher levels of deprivation. However, the magnitudes of the deviations in Havering were very small.

**Table 2:** Correlation coefficients for the difference between predicted and gold standard scores versus the average of predicted and gold standard scores.

**District**	**Pearson's correlation coefficient (p value) for difference between predicted and gold standard scores versus average of predicted and gold standard scores**	
	**Postcode-linked score – gold standard score comparison**	**Modelled score – gold standard score comparison**
	
Doncaster	0.57 (p < 0.001)	-0.22 (p = 0.15)
Havering	0.65 (p < 0.001)	-0.49 (p < 0.001)
Warrington	0.57 (p = 0.002)	0.03 (p = 0.88)
All three districts combined	0.58 (p < 0.001)	0.04 (p = 0.68)

The differences between the postcode-linked score predictions and gold standard scores were greater than the differences between the modelled and gold standard scores as shown by the wider 95% agreement limit intervals on plots 5 to 8. For Doncaster and Havering, and for the combined analysis, this difference was significant (Fligner-Killeen test for homogeneity of variances p < 0.001, see Table 3).

**Table 3:** Fligner – Killeen test for homogeneity of variance between the postcode linked predictions – gold standard scores, and the modelling method predictions – gold standard scores.

**District**	**Fligner-Killeen test for homogeneity of variance between the two predictive methods**
Doncaster	χ^2 ^= 17.21, df = 1, p < 0.001
Havering	χ^2 ^= 32.56, df = 1, p < 0.001
Warrington	χ^2 ^= 1.90, df = 1, p = 0.167
All three districts combined	χ^2 ^= 52.39, df = 1, p < 0.001

### Sensitivity analysis using different weightings for main and branch surgeries

The predicted scores in the above results were calculated using a simple equal weighted average for the scores predicted for the main and any branch surgeries. We examined the performance of the model (for those practices with branches) under a range of alternative main-branch surgery weightings in a sensitivity analysis. As the weighting given to the main surgery increased from an equal share towards 100%, the mean difference between the predicted scores and the "gold standard" scores deviated away from zero, suggesting an increasingly biased prediction. In a secondary analysis comparing results for single site versus multi site practices across all three districts, the model performed no worse when predicting scores for practices with more than one site (mean difference between modelled scores and gold standard scores for single site practices: 0.25 IMD points, 95% CI -1.25 to 1.75; for multi-site practices: 0.39, 95% CI -0.35 to 1.14).

## Discussion

### Main results

This study has shown how a fairly simple model of the distribution of registered patients around a general practice can be used to predict a practice population-weighted area-based measure of socioeconomic deprivation. This method appeared superior to the common practice of linking the practice postcode alone to the deprivation score assigned to the area in which the practice is located. There were some differences in the ability of the model to accurately predict scores between the three districts. One reason for this may be the existence of groups of practices that, although they are in close geographical proximity, attract patient populations from quite different socioeconomic or ethnic backgrounds. This is certainly the case for Doncaster and will reduce the predictive accuracy of the model in an unpredictable manner. There may also be in some districts practices that draw their patients from a particular subgroup of the population, for example, students or asylum seekers. A deprivation score for such a practice would be difficult to predict using any method that did not have access to data regarding the registered patient population.

### Strengths

Our proposed modelling method may be particularly useful for researchers who need a measure of the socioeconomic deprivation experienced by a general practice population, but who do not have access to patient level spatially referenced data. The method was relatively straightforward to execute using basic GIS functions within a commonly used mapping package, MapInfo Professional 8.0 [11]. Although we obtained Census and postcode data using the academic sources Casweb [18] and Edina [21], equivalent data sets are available free within the NHS [27,28].

### Limitations

The data used to construct the model were derived from 55 practice sites in Rotherham, and the model will therefore be most accurate when used to predict scores for practices whose geographical distribution of patients is similar to that found in Rotherham. The method makes a number of assumptions: firstly that registered patients will be equally distributed in all directions around a practice, which is unlikely to hold true in all cases, and especially not so at natural or administrative boundaries; secondly that the same spatial distribution of patients exists around urban and rural practices and around practices regardless of their size. Again, this is a simplification of a complex real-world situation. Thirdly we assumed that all patients lived within the PCT in which the practice was located. This will not always be the case, particularly at PCT boundaries. However, a PCT would have to make the same assumption in calculating a patient weighted score using individual level spatially referenced data. This is because PCTs routinely have access only to individual data for their resident population, rather than the whole population registered with their contracting GPs. Despite these limitations the model performed well at predicting scores in three other districts within the UK.

We assumed, where a practice had one or more branch surgeries, that equal numbers of patients attended each surgery site. This may or may not be true, and intuitively it may seem that more patients would attend a main practice than would attend a branch practice. The number of patients registered at a branch surgery is not routinely recorded outside of the practice, and we could find no literature from which to estimate an average distribution. Perhaps surprisingly, we found in our sensitivity analysis that an assumption of an equal distribution of patients between each surgery site led to a less biased model prediction than did assuming that a greater proportion of patients attended the main site than any branch sites. Under the assumption that equal numbers of patients attended each surgery site we found that the model performed just as well when predicting deprivation scores for multi-site practices as it did for single site practices. It would be straightforward, however, for the model to be applied assuming a different weighting between main and branch surgeries if there were particular reasons (e.g. local knowledge of practice characteristics) to support this.

The Index of Multiple Deprivation 2004 used in this model is an area-based measure. It is derived from variables measured at the group level (for example the proportion of population in a household receiving the Income Support welfare benefit), and applies therefore to the group, rather than any single individual. An individual living within the group may experience a quite different level of deprivation. "Well off" people live in areas of high deprivation, and vice versa. Associations seen between area-based deprivation and other variables, such as mortality or disease prevalence, may or may not apply at the individual level, and assuming it does apply is known as the "ecological fallacy". Despite this limitation, area level associations are important whether they reflect individual level associations or not, and indeed some variables such as social cohesion, are meaningfully analysed only at a group level.

A second potential limitation of using the IMD 2004 when exploring associations between deprivation and practice level health related measures is the inclusion within IMD 2004 of a "Health Deprivation and Disability Domain". This could, in theory, lead to an inevitable correlation between the deprivation measure and the health outcome variable of interest, a problem known as "mathematical coupling". However, removing the health domain from IMD 2004 has been shown to make little practical difference [29]. An alternative course of action would be to use, say, the IMD 2004 income domain alone to construct a practice level measure of deprivation. Our model would be equally useful, since it is essentially a weighting method that can be applied to any variable linked to a small area.

## Conclusion

A simple GIS based model, based on an assumed exponential decay distribution of patients around a general practice, can be used to predict a practice population-weighted area-based deprivation measure. Our modelled measure had better agreement with the population-weighted measure than did a postcode-linked measure alone. Our model may also avoid a systematic underestimation of IMD score in less deprived areas, and a systematic overestimation of scores in more deprived areas, that was seen when using postcode-linked scores. This method may therefore be of use to researchers who do not have access to patient level spatially referenced data.

## Competing interests

The author(s) declare that they have no competing interests.

## Authors' contributions

MS conceived the study, analysed the data and wrote the manuscript. RM refined the study question and critically revised the manuscript. TP and PF provided advice regarding the analysis and critically revised the manuscript. All authors have read and approved the final manuscript.
